# Drink wise, age well; reducing alcohol related harm among people over 50: a study protocol

**DOI:** 10.1186/s12889-019-6525-7

**Published:** 2019-02-28

**Authors:** J. L. Seddon, S. Wadd, E. Wells, L. Elliott, I. Madoc-Jones, J. Breslin

**Affiliations:** 1 Substance Misuse and Ageing Research Team, Tilda Goldberg Centre for Social Work and Social Care, University of Bedfordshire, Park Square, Luton, Bedfordshire, LU1 3NJ UK; 2 Drink Wise, Age Well Programme, Furnival House, 48 Furnival Gate, Sheffield, S1 4QP UK; 3 School of Health and Life Sciences, Glasgow Caledonian University, Glasgow, G4 0BA UK; 4 Glyndŵr University, Wrexham, Wales LL11 2AW; 5 Drink Wise, Age Well Programme, 34 Argyll Arcade, Buchanan Street, Glasgow, G2 8BD UK

**Keywords:** Alcohol use, Drinking, Older adults, Older people, Alcohol intervention

## Abstract

**Background:**

Evidence suggests that the use of alcohol among older adults (defined as those aged 50+) has increased in recent years, with people aged 55–64 now more likely to exceed the recommended weekly guidelines than any other age group.

**Methods/ design:**

This is a quasi-experimental study with a before-after design. A postal questionnaire will be sent to 76,000 people aged 50 and over registered with a general practice in five different ‘demonstration’ (intervention) and control areas in the UK. Multiple interventions will then be delivered in demonstration areas across the UK. At the end of the programme, a postal questionnaire will be sent to the same individuals who completed it pre-programme to establish if there has been a reduction in alcohol use, at-risk drinking and alcohol related problems. Qualitative interviews with clients and staff will explore how the interventions were experienced; how they may work to bring about change and to identify areas for practice improvements.

**Discussion:**

This study protocol describes a multi-level, multi-intervention prevention-to-treatment programme which aims to reduce alcohol-related harm in people aged 50 and over.

## Background

The World Health Organisation has identified alcohol-related harm among older adults as an increasing concern [1]. In the UK, evidence suggests that alcohol use among older adults is increasing [2–6] and people aged 55–64 are more likely to exceed the recommended weekly guidelines than any other age group [3, 5].

The use of alcohol, even in small amounts can be problematic for older people as they may metabolise and excrete alcohol more slowly [7], and alcohol can accelerate and exacerbate the onset of conditions associated with ageing such as falls [8] and cognitive impairment [9]. The use of alcohol alongside other medication can also result in adverse interactions, such as raising blood alcohol levels, altering the metabolism of many drugs, reducing the efficacy of medication and exacerbating medication side effects [10].

Alcohol related hospital admissions in England were estimated at 1.1 million in 2015/2016; 45% of these were for individuals aged 55–74 years of age [11]. In addition, in 2016, the rate of alcohol specific deaths in the UK was highest among those aged 55–64 years, with a significant increase in alcohol related mortality among older people between 2001 and 2016 [12].

Changes in lifestyle and life transitions that occur as people age, such as deteriorating health, bereavement, and retirement may precipitate a change in drinking behaviour, and it is estimated that 1 in 3 older people with an alcohol problem begin drinking in later life [13]. Evidence also suggests that many older people with alcohol problems may not seek help due to associated feelings of shame and stigma [14].

In 2014 the median age of the UK population exceeded 40 for the first time. It is expected that over 70% of UK population growth between 2014 and 2039 will be in the over 60 age group, with the number of people aged 60+ increasing from 14.9 to 21.9 million [15]. With an increasing ageing population and evidence of increased drinking among older adults, action is urgently needed to reduce alcohol-related harm in older adults.

There have been very few interventions designed to address alcohol use among older adults, and these have typically been conducted in primary care settings [16–20]. The current project: *Drink Wise, Age Well* (DWAW), however, is a community based, multi-level, multi-intervention prevention-to-treatment programme which aims is to reduce alcohol-related harm in people aged 50 and over.

The DWAW project has four main aims:

i) To raise awareness of the issue of alcohol misuse among people over 50, change attitudes, reduce stigma, convey harm reduction messages and influence community norms about the use of alcohol.
ii) To increase individual and community resilience to alcohol problems in people over 50 and to reduce hazardous, harmful and dependent drinking and related harm in this age group.
iii) To increase the extent to which community service providers and employers who have regular contact with people over 50 are able to recognise and respond to risky drinking.
iv) To develop a body of evidence on how to prevent alcohol misuse in people over 50 which will inform future prevention work in the UK.

## Methods/design

This is a quasi-experimental study with a before-after design which will compare drinking behaviour in a sample of people aged 50 and over in the demonstration (intervention) areas with that of people aged 50 and over in control areas (no intervention). Multiple interventions will be delivered over a period of five years in five demonstration areas across the UK (England: Sheffield and Devon, Scotland: Glasgow, Wales: Cwm Taf, and Northern Ireland: Western Trust Area). These areas have been selected as they present specific challenges (e.g. high levels of alcohol use; deprivation; high proportion of marginalised groups that are under-represented in alcohol services). Control areas have been selected on the basis that they are similar to the demonstration areas in terms of population size and characteristics, these are: Derby and Lincolnshire (England), Dundee (Scotland), Betsi Cadwaladr (Wales) and the Southern Trust Area (Northern Ireland).

The rationale for delivering multiple activities in demonstration areas is that the activities will complement and reinforce each other. A broad-based approach reflects the finding from general population studies that risk for alcohol problems exist along a continuum [21], and targeting only alcohol-dependent individuals or those with a history of alcohol-related problems is not sufficient. The DWAW programme aims to reduce alcohol related harm using a community based approach. Peer facilitators and volunteers will be recruited to deliver aspects of the programme to strengthen community capacity and increase sustainability.

### Interventions

**Fig. 1:**
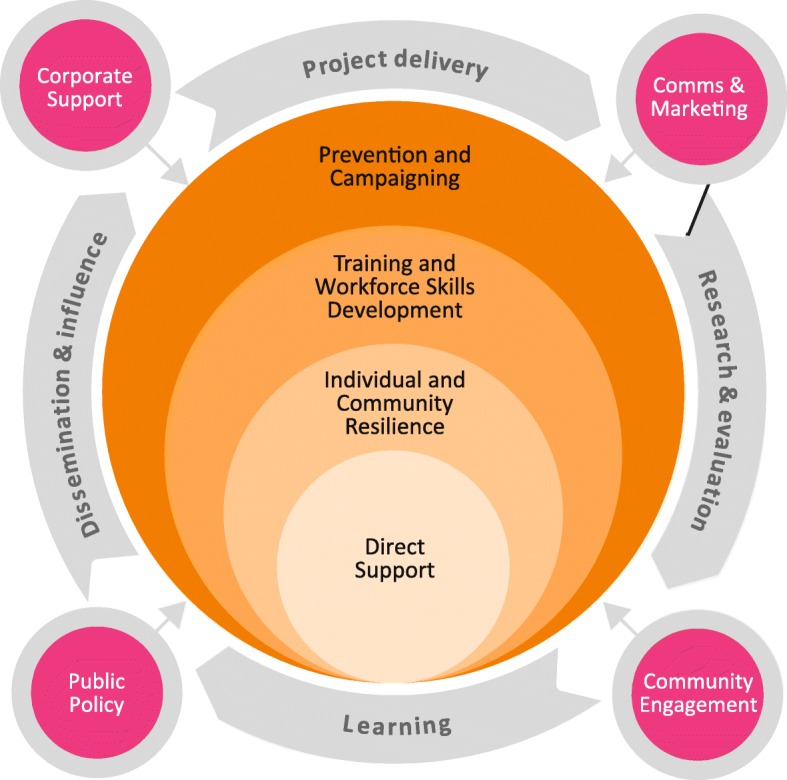
Drink Wise, Age Well Programme Model

The range of interventions delivered as part of the programme broadly fall into one of four main categories: i) increasing awareness, tackling public stigma and providing information and advice, ii) training and skills development, iii) increasing resilience, iv) structured support for people over 50 experiencing alcohol problems. The DWAW programme is shown in Fig. 1.

#### i). Increasing awareness, tackling public stigma and providing information and advice

Many older adults may be unaware of the effects that higher-risk drinking could be having on their lives such as poor sleep [22], cognitive impairment [23], and adverse interactions with medication [10, 24].

To raise awareness of alcohol related harm among people aged 50+, advertisements in local media (e.g. newspapers, television, radio) and healthcare settings (e.g. GP surgeries, hospital waiting rooms) will promote the DWAW programme and provide information about the effects of alcohol use among older people. Information will also be disseminated through workshops and public information stalls. People attending workshops and engaging at public information stalls will also be offered screening using the AUDIT-C [25] and a brief intervention for alcohol use based on the FRAMES approach [26]. A telephone helpline, webchat service, health promotion website and a series of marketing materials including a film to tackle stigma will be developed to further increase awareness and provide advice.

#### ii). Training and skills development

Older people frequently have contact with service professionals such as health and social care practitioners, carers, and bereavement support organisations who are well placed to identify and respond to problematic alcohol use. The DWAW programme will provide alcohol awareness training to people working in the health and social care field; a series of training manuals will be developed to ensure consistency, and organisations will be able to choose from half a day of training up to two full days of training. Through training and closer formal and informal partnership working it is hoped that a greater number of older people will benefit from earlier identification of problematic alcohol use and pathways into appropriate support and treatment will be reinforced.

Substance misuse practitioners working in mixed-age services will also be trained to work in an age-sensitive manner appropriate for older people, by taking account of the unique physical, psychological, social and cognitive features of ageing.

#### iii). Increasing resilience

Evidence suggests that alcohol use in older adults may increase as a result of stress and loss of role and identity [27]. A six-week resilience group work course, known as *Live Wise, Age Well* will be delivered in a variety of settings (e.g. workplaces, community settings). This will focus on the use of alcohol among older adults, stress management, coping skills, relaxation techniques and mindfulness. It will also cover areas of well-being such as diet, sleep and exercise. Clients who take part in the six week resilience course will complete assessments at entry and discharge. The assessment will collect data on participant socio-demographics, alcohol use (AUDIT-C) [28], resilience (Brief resilience scale) [29], and mental well-being (Warwick-Edinburgh Mental Wellbeing Scale) [30].

Community-level social activities, skills workshops and social events will also be delivered and will include alcohol related information. The aim is that these will eventually become self-sufficient and run independently of DWAW.

The resilience course and social activities will try to target older adults most at risk of increasing their alcohol use as they age (e.g. people experiencing stressful life events, or with a previous history of alcohol misuse) but will be open to all older adults in order to build resilience at a community level.

#### iv). Structured support for people over 50 experiencing alcohol problems

Research demonstrates that substance use services designed for older adults may be more acceptable to older people than mixed aged services [14] and may be linked to better outcomes than mixed-age services [20, 31–35]. Structured support for alcohol use specifically designed to meet the needs of older adults will be provided. This will include age sensitive assessments, screening for cognitive impairment, interventions adapted to the needs of older adults (e.g. focused on life stage issues) and peer support groups for people over 50. Family support services including the 5-step method [36] will also be offered. Multi-agency working with other health and social care providers will help to strengthen the referral pathways to the service.

Clients will complete assessments at entry, discharge and 6 months post-discharge. The assessments will collect data on socio-demographic details, alcohol use (e.g. AUDIT [25], days used alcohol in last 4 weeks, units consumed on a typical day), physical health, depression (PhQ-9) [37], anxiety (GAD-7) [38], mental well-being (Warwick-Edinburgh Mental Wellbeing Scale) [30], and cognitive functioning using the Montreal Cognitive Assessment [39].

### Study monitoring and review

To ensure that key components of the interventions are being delivered and evaluated the project advisory group will be convened at 3-month intervals, involving the principal investigators and all researchers.

### Evaluation

The overall evaluation strategy for the DWAW programme has been designed to assess the combined impact of all the interventions on alcohol consumption and at-risk drinking in people aged 50 and over. This will be done using a large-scale postal survey. Qualitative interviews with DWAW staff and participants will further explore how the interventions were received and how they may have contributed to change in alcohol use for people aged 50 + .

#### Quantitative survey data of people aged 50 and over

An anonymous postal questionnaire will be sent to 76,000 people aged 50 and over registered with a general practice in the demonstration and control areas. The questionnaire will collect information on alcohol use (AUDIT) [25], alcohol use frequency, units consumed, reasons for use, knowledge of alcohol units etc.), health (SF-12) [40] and healthcare use. All participants will be provided with a written study information sheet and it will be made clear that completion and return of the questionnaire implies consent to take part.

The questionnaires will be completed in two waves; the first wave will be prior to implementation of the programme (year 1), the second wave will be at the end of the programme (year 5). The questionnaire will be sent to the same individuals at both time-points linked by a unique identification number. Personally identifiable data will not be collected as part of the questionnaire. To recruit participants, 3 GP practices in each demonstration and control area will use the Health Informatics Centre at the University of Dundee to send a questionnaire, postage paid envelope and covering letter to all over 50’s on their practice lists asking them if they would like to participate in the study. In Scotland, Wales and England, a GP will check the list of participants beforehand for suitability (e.g. patients nearing the end of life or who don’t have the capacity to consent will be removed). In Northern Ireland, this task will be carried out by a Clinical Research Nurse from Northern Ireland CRN (Primary Care) in conjunction with a GP. Patient names and addresses will not be made available to the research team and only the research team will have access to the data.

Questionnaires will take approximately 15 min to complete. Participants will be able to return the completed questionnaires directly to the research team in a pre-paid envelope or complete it on their mobile phone, tablet or PC. We anticipate a 35% response rate. Returned questionnaires will be electronically scanned and stored on password protected computers. If participants do not complete a questionnaire in Wave 1 (pre-programme), they will not be sent a questionnaire in Wave 2 (post-programme).

At the end of the programme (year 5) the questionnaire will be sent to all participants who completed the questionnaire at wave 1. A written reminder will be sent if participants have not responded within two weeks, with a second reminder sent after 1 month.

The aim of the questionnaire survey is to establish if there has been a reduction in alcohol consumption, at-risk drinking and alcohol-related problems in over 50’s in the demonstration areas, as well as measuring programme reach and awareness of campaign messages.

- *Sample size & analysis plan- survey data*

The sample size calculation is based on detecting a reduction of 2 alcohol units per week in the intervention groups (i.e. demonstration areas). For the purposes of this calculation this will be re-phrased as a difference in the average reduction between the two groups, if there is no identified difference in the average consumption of the control group then these two definitions are comparable.

An estimate of overall consumption in the over-50’s including the 17% of over 50’s who abstain or drank nothing in last year comes from the General Lifestyle Survey 2010 [42] and is given as a mean of 10.7 with an SD of 17.4, so the effect size will be 2/17.4, rounded to approximately 0.1. Consulting Cohen [41], Table 2.4.1 (p55), and taking a power of 0.80, type I error of 0.5, and an effect size of 0.1 gives a sample size of 1571 for each group.

A complete or partial response was obtained in the General Lifestyle Survey 2010 [42] for of the order of 70%, with design effects generally of the order or 1.2 to 1.3 or less. Thus, allowing for non-response, design effects and males and females separately results in a sample size of 5500 per group (i.e. demonstration or control area).

Regression modelling will be used to assess the differences between the intervention and comparison groups at baseline and post intervention. Due to the quasi-experimental, serial cross-sectional design, difference-in-differences analyses will be conducted using logistic and linear regression for dichotomous and continuous outcomes, respectively.

#### Qualitative evaluation

The qualitative evaluation of DWAW aims to explore how interventions are experienced; how they may work to bring about change and to identify areas for practice improvements. The qualitative evaluation will also explore if DWAW is meeting the needs of minority groups and over 50’s experiencing different life stages and circumstances.

Semi-structured interviews will be conducted with people who have received structured alcohol support or taken part in the DWAW resilience course. Focus groups will be conducted with recipients of the DWAW group interventions (e.g. peer support groups, social activity groups). Participant case studies using photo elicitation will also be completed in each of the demonstration areas to explore the relationship with alcohol and any change in use. Photo elicitation involves the participant making a visual record of their lives whilst they are engaged with DWAW; this is then used to generate discussion during interview. Focus groups will be conducted with DWAW staff throughout the programme, and individual interviews will be conducted with stakeholders (professionals involved in multi-agency working with DWAW). All interviews will be audio-recorded and will take no longer than one hour. Interviews will be transcribed verbatim and will be analysed using a framework approach [43].

The findings of this study will be disseminated at both national and international conferences and published in peer-reviewed journals.

## Discussion

Alcohol use prevention and intervention programmes specifically designed for older adults are rare, and interventions designed for mixed-age groups may fail to take into account the unique physical, psychological and cognitive features of aging. To our knowledge, DWAW is the first multi-level programme of its kind anywhere in the world for people aged 50 and over.

Using a community based approach, DWAW aims to reduce alcohol consumption, at-risk drinking and alcohol-related problems for people aged 50 and over. The programme will also increase awareness of the impact of alcohol use in older people, reduce stigma, improve identification of problematic alcohol use, and provide holistic support to people with alcohol related issues in an age-sensitive manner.

The model of DWAW will empower communities to become part of the solution and seeks to mobilise assets within communities. This community based approach is not only an efficient use of resources; evidence suggests that public health programmes are more likely to be effective if the beneficiaries are active contributors and not passive recipients [44]. It is hoped that the model of community empowerment and increasing resilience at an individual and community level will result in a lasting impact that persists beyond the life of DWAW.

## Abbreviation

DWAW: Drink Wise, Age Well
